# Complete Genome Sequence of *Clostridium estertheticum* DSM 8809, a Microbe Identified in Spoiled Vacuum Packed Beef

**DOI:** 10.3389/fmicb.2016.01764

**Published:** 2016-11-11

**Authors:** Zhongyi Yu, Lynda Gunn, Evan Brennan, Rachael Reid, Patrick G. Wall, Peadar Ó. Gaora, Daniel Hurley, Declan Bolton, Séamus Fanning

**Affiliations:** ^1^UCD-Centre for Food Safety, School of Public Health, Physiotherapy and Sports Science, School of Biomedical and Biomolecular Science, University College DublinDublin, Ireland; ^2^Teagasc Food Research CentreDublin, Ireland

**Keywords:** blown pack spoilage, *Clostridium estertheticum*, vacuum packed beef, whole genome sequencing, food quality

## Abstract

Blown pack spoilage (BPS) is a major issue for the beef industry. Etiological agents of BPS involve members of a group of *Clostridium* species, including *Clostridium estertheticum* which has the ability to produce gas, mostly carbon dioxide, under anaerobic psychotrophic growth conditions. This spore-forming bacterium grows slowly under laboratory conditions, and it can take up to 3 months to produce a workable culture. These characteristics have limited the study of this commercially challenging bacterium. Consequently information on this bacterium is limited and no effective controls are currently available to confidently detect and manage this production risk. In this study the complete genome of *C. estertheticum* DSM 8809 was determined by SMRT^®^ sequencing. The genome consists of a circular chromosome of 4.7 Mbp along with a single plasmid carrying a potential tellurite resistance gene *tehB* and a Tn*3-*like resolvase-encoding gene *tnpR*. The genome sequence was searched for central metabolic pathways that would support its biochemical profile and several enzymes contributing to this phenotype were identified. Several putative antibiotic/biocide/metal resistance-encoding genes and virulence factors were also identified in the genome, a feature that requires further research. The availability of the genome sequence will provide a basic blueprint from which to develop valuable biomarkers that could support and improve the detection and control of this bacterium along the beef production chain.

## Introduction

*Clostridium estertheticum* is a Gram-positive, spore-forming bacterium that is recognized as a causative agent of blown pack spoilage (BPS) in vacuum packed beef products (Bolton et al., 2015). Such events, which may occur as soon as 4 weeks following storage, can lead to economic losses for a meat producer. Despite the implementation of good manufacturing practice (GMP), BPS, including the production of carbon dioxide along with a metallic sheen on the meat, may occur and meat spoiled in this way has no commercial value (Broda et al., 1996).

Besides the associated economic loss, controlling BPS continues to be challenging. One strategy deployed is to decontaminate the abattoir with peroxyacetic acid, a known corrosive chemical treatment that exerts a negative impact on the fabric of the production site. Despite these cleaning protocols, spores can remain unaffected, only to germinate at a later stage. The potential occurrence of BPS is one of the main reasons that beef abattoirs cannot adopt the *hot/warm boning technique*, a measure that would support improvements in beef quality, at a reduced cost through better production methods (Bolton et al., 2015).

Several bacterial species of the genus *Clostridium* have been recognized in BPS episodes. These include *C. estertheticum. Clostridium gasigenes*, and *Clostridium ruminantium*, all of which can be difficult to culture and study in the bacteriology laboratory. Several weeks or in some cases even months are necessary to obtain a workable culture. Thus, even the most routine laboratory protocols can be frustrating to implement and to troubleshoot. Phenotype-based approaches for the detection of these bacteria are not a feasible option. When the existing literature was examined, this view was supported by the fact that a small number of molecular-based detection strategies have been developed, mainly targeting the 16S rRNA genes, and which are applied with variable success.

The availability of the genome sequence would begin to provide a basis upon which to extend our knowledge of these bacteria and these data could be expected to support the development of improved diagnostic biomarkers. Determining the complete genome of one or more etiological agents of BPS would provide a suitable reference, from which to identify candidate gene(s) that could be assessed for their utility as diagnostic markers in the development of new PCR-based detection methods. As a step toward this objective, in this paper we report the whole genome sequence of *C. estertheticum* DSM 8809, an important etiological agent identified in episodes of BPS.

## Materials and Methods

### Bacterial Culture

*Clostridium estertheticum* subsp. *estertheticum* DSM 8809 was acquired from Leibniz Institute DSMZ-German Collection of Microorganisms and Cell Cultures. The bacterium was grown in brain–heart infusion (BHI) broth under anaerobic conditions at 8°C for at least 2 weeks prior to the purification of genomic template DNA (gDNA).

### DNA Purification and Sequencing

Genomic DNA was purified from *C. estertheticum* DSM 8809 using a phenol–chloroform method, that was modified from those previously reported and used with *Clostridium difficile* (Wren and Tabaqchali, 1987; Bouillaut et al., 2011). In brief, 5 ml grown cultures of bacteria were harvested by centrifuging at 10,000 × *g* for 2 min. The resulting pellet was resuspended in 1 ml TE buffer (10 mM Tris-HCl and 1 mM EDTA, at pH 8). It was then centrifuged again at 10,000 × *g* for 2 min, and the pellet re-suspended in 200 μl genomic DNA solution (consisting of 34.23 g sucrose/100 ml TE, filter sterilized) to which 50 μl freshly prepared lysozyme (50 mg/ml) was added. This mixture was incubated for 90 min at 37°C. Following this step, 100 μl 20% [w/v] sarkosyl and 15 μl RNase A (10 mg/ml) was then added incubated for a further 30 min at 37°C. Fifteen μl proteinase K (10 mg/ml) was then added and the solution was incubated for another 30 min at 37°C. In the final step of the purification protocol, TE buffer was then added to bring the final volume to 600 μl. The solution was then mixed with 600 μl 25:24:1 phenol/chloroform/isoamyl alcohol and centrifuged at 17,000 × *g* for 10 min. and the resulting upper aqueous phase transferred to a clean microfuge tube. All of the above steps following the addition of 600 μl phenol/chloroform/isoamyl alcohol were repeated twice to achieve a more pure gDNA template for sequencing. Upon completion of the final step, gDNA was precipitated by adding 50 μl 3 M sodium acetate (pH 5.2) and three volumes of cold 95% [v/v] ethanol. This solution was maintained at -20°C for at least 1 h. After that, the solution was centrifuged at 17,000 × *g* for 5 min. The pellet was washed with 500 μl 70% [v/v] ethanol, air-dried before being finally re-suspended in 100 μl TE buffer and allowed to dissolve at room temperature overnight.

Library preparation using the above purified gDNA and the subsequent sequencing was carried out commercially by the Centre for Genomic Research at the University of Liverpool. In brief, gDNA was sheared to approximately 10-kbp and then sequenced on a Pacific Biosciences (PacBio) RS II instrument (P4/C2 chemistry) with two single molecule real-time (SMRT^®^) cells. This approach provides for the sequencing of longer reads and is suitable when a complete genome sequence is required.

### Genome Assembly

*De novo* assembly of the read data obtained was completed by SMRT^®^ Analysis using the Hierarchical Genome Assembly Process (HGAP) 2.0 protocol (Chin et al., 2013). Minimum seed read length was set at 12,000 bp for PreAssembler v2. Expected genome size was set to 4,800,000 bp [as indicated by an HGAP 3 trial run (data not shown)]. This approach resulted in two contigs (with mean QV score at 48.8 and 47.7, respectively) that were subsequently circularized by Circlator 0.16.0 (Hunt et al., 2015) using corrected sub-reads generated by HGAP 2 (with 6,000 minimum seed read length). The ‘RS_Resequencing.1’ protocol on SMRT^®^ Analysis was then used to align raw reads to the circularized genome for an improved consensus.

### Validation of the Genome Structure by Selected PCR Sampling of the Chromosome and Plasmid

The coverage information of the genome was visualized by SMRT^®^ View which provided data on the sequence at intervals of 10,000 bp. The coverage obtained after sequencing for each base pair was determined by using the ‘genomecov’ function in ‘BEDtools’ (Quinlan and Hall, 2010) a strategy that would indicate regions that may contain spikes, related to lack of sequence conformity. PCR primers were then designed with the aid of ‘Primer3’ (Koressaar and Remm, 2007; Untergasser et al., 2012) to sample randomly around the chromosome and plasmid and to support the consensus assembly. As before, all amplicons were sequenced by traditional Sanger sequencing protocols. The resulting sequences were then merged by ‘Contig Assembly Program’ (CAP) in BioEdit (Hall, 2011).

### Plasmid DNA Extraction

Plasmid DNA was purified from *C. estertheticum* DSM 8809 using an alkali lysis method (Bimboim and Doly, 1979). The resulting DNA pellet obtained from 5 ml bacterial culture was re-suspended in 200 μl of a solution containing 50 mM glucose, 10 mM Tris-HCl pH8, 10 mM EDTA, 10 mg/ml lysozyme and 10 mg/mL RNase A and incubated for 1 h at 37°C. To this mixture was added 400 μl 0.2% [w/v] NaOH and 1% [w/v] SDS. Three-hundred μl 7.5 M ammonium acetate and 300 μl chloroform as then added and incubated on ice for 10 min. The solution was centrifuged and the supernatant was transferred to a new tube containing 200 μl chilled precipitation buffer (30% PEG200, 1.5 M NaCl) and incubated on ice for 15 min. The purified plasmid DNA was recovered after centrifugation at 10,000 × *g* and the pellet was finally re-suspended in 50 μl 1x TE buffer.

### Gene Identification Using BacMet/VFDB

The raw assembly was submitted to Prokka (Seemann, 2014) to identify and annotate genes. The output was used to search for similar matches in BacMet (Pal et al., 2014) and virulence factor database (VFDB) (Chen et al., 2011) databases using BLASTP (Camacho et al., 2009). Only alignments with over 70% coverage of the genes from these databases were included in the final annotation. These results were then filtered according to percentage of identity (threshold 50%) and differences in gene length (50 bp).

### KO Mapping

Metabolic pathways were reconstructed for each of *C. estertheticum* DSM 8809, *Clostridium botulinum* Hall A and *Clostridium perfringens* 13 using ORFs as identified by RAST annotation (Aziz et al., 2008) and the KEGG Automatic Annotation Server (Kanehisa and Goto, 2000). Amino acid sequences for each ORF were provided and orthologs were assigned using the bi-directional best hit (BBH) method. The representative gene data set “for Prokaryotes” was used in addition to the inclusion of manually annotated organisms *C. botulinum* Hall A (cbh) and *C. perfringens* 13 (cpe) for the mapping of all three isolates.

## Results

### Whole Genome Sequencing of *Clostridium estertheticum* DSM 8809

Several DNA extraction protocols reported by previous researchers were assessed for their suitability to provide purified gDNA of sufficient concentration and quality for SMRT^®^ sequencing. Following several attempts the phenol–chloroform method was considered to be suitable and capable of yielding higher qualities of gDNA that would meet the SMRT^®^ sequencing requirements (data not shown). This enabled the purification of a good quality sequencing template that resulted in 175,417 reads and 3,526,834,644 bases being produced following SMRT^®^ sequencing.

**FIGURE 1:**
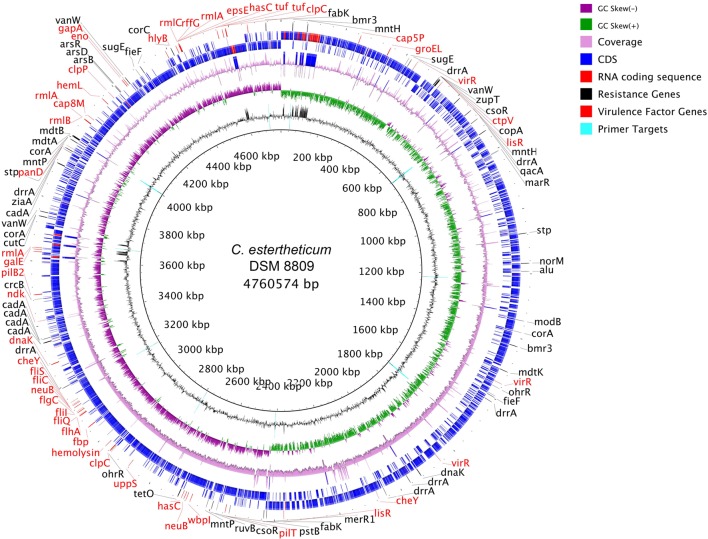
**Representation of the completed chromosome of *Clostridium estertheticum* DSM 8809.** Each ring, beginning from the inside out represents the following features: GC content (black) with PCR targets marked as aqua-colored; GC skew (green and purple); sequencing coverage (pink); genes encoded on the negative strand (blue for CDS, red for RNA); genes encoded on positive strand (blue for CDS, red for RNA); biocide resistance genes (shown in black colored font), metal resistance genes (also shown in black colored font), and virulence factor genes (shown in red colored font).

**FIGURE 2:**
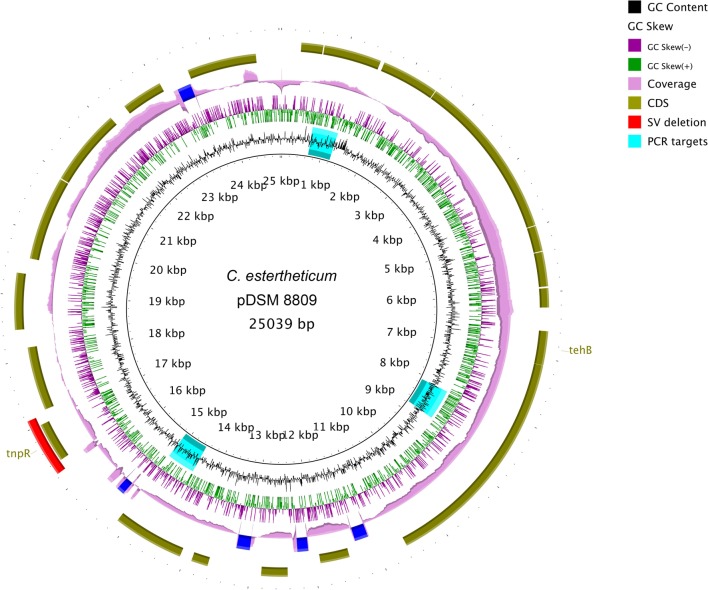
**Complete sequence of a 25-kbp plasmid pDSM8009 contained in *C. estertheticum* DSM 8809.** Each ring, beginning from the inside out represents the following features: GC content (black) with PCR targets marked as aqua-colored; GC skew (green and purple); sequencing coverage (pink); genes encoded on the negative and positive strand (dark olive green); Structural variant (SV) deletion (red). A predicted tellurite resistance gene *tehB* and a putative Tn*3*-like transposon resolvase *tnpR* are located on the positive strand. The SV deletion is also located at the position of *tnpR* gene.

**FIGURE 3:**
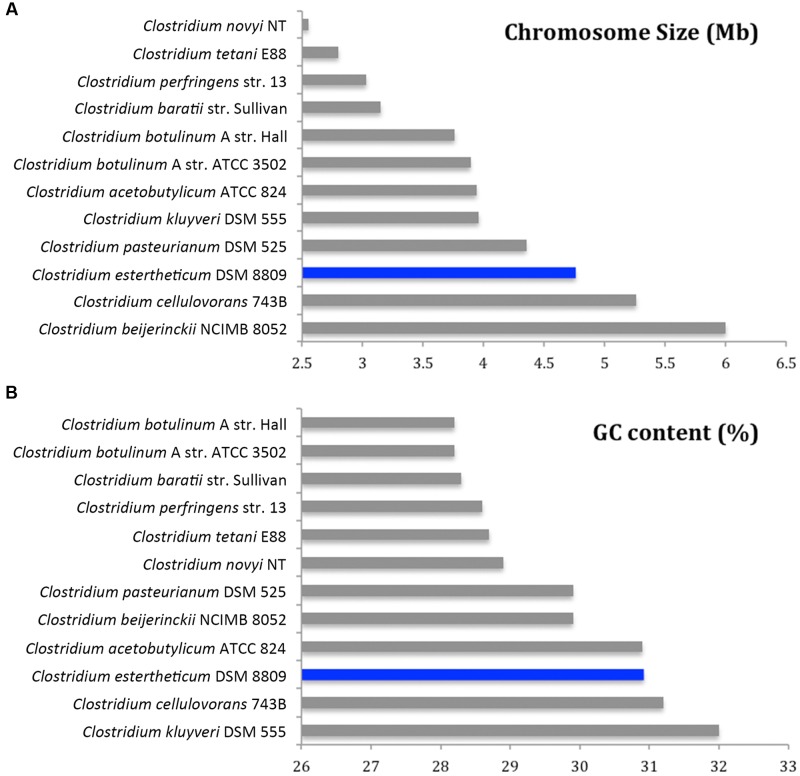
**Comparative analysis of genome size** **(A)** and GC content **(B)** of *C. estertheticum* DSM 8809 with a selection of other available genomes representative of *Clostridium* species.

*De novo* assembly with HGAP3 using the default settings produced 11 polished contigs (data not shown). After optimizing of the assembly protocol two contigs were produced, that constituted the complete genome sequence of *C. estertheticum* DSM 8809 (GenBank accession number CP015756 and CP015757). The genome consisted of two circularized DNA molecules that included, a 4,760,574 bp chromosome (**Figure 1**) and a 25,039 bp plasmid pDSM8809 (**Figure 2**). **Figure 3** provides a comparison of the general features of the genome of *C. estertheticum* DSM 8809 with other species of this genus. The chromosome of the latter is one of the largest in the genus (**Figure 3A**) whilst its GC content of 30.92%, was lower *than C. cellulovorans* and *C. kluyveri*, both of which are at the upper end of the range (**Figure 3B**).

**FIGURE 4:**
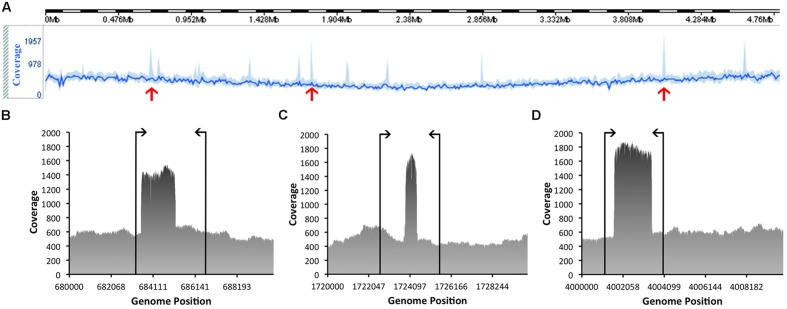
**Coverage information related to the assembly of the genome of *C. estertheticum* DSM 8809.** The line in bright blue **(A)**, located toward the top of the figure, represents the average sequence coverage of each window, denoted as **(B–D)** consisting of 10,000 bp and the area in lighter blue indicates the maximum and minimum coverage of the window. Spikes marked with red arrows highlight those regions referred to in the text and which were subsequently checked by PCR to validate the true nature of the consensus sequence. Annealing positions of the PCR primers are indicated by vertical lines, as shown for **(B–D)**.

The quality of these data was then assessed. Based on this evaluation, the genome has a mean coverage of 522.14-fold. Analyses of the coverage graph (**Figure 4**) highlighted a number of spikes that reflected areas with significantly higher coverage. These spikes may have resulted from the merging of repeat regions contained within the genome. In order to validate the genome sequence, three sets of PCR primers were designed, each flanking a coverage spike (see **Table 1**; **Figure 4**). The size of the amplicons obtained after PCR were in complete agreement with the predicted size from the genome, confirming the accuracy of the assembly (data not shown).

**Table 1:** Oligonucleotide primer sequences designed to validate the genomic sequence of *Clostridium estertheticum* DSM 8809.

Forward (F) and reverse (R) primer Sequence (5′→′)	Location/position (bp)	Location/sequenced region	Identity
**F-**ATG CAG GCA CAA CAA CCA TT	Chromosome	Chromosome	100%
**R-**AAT GCC TTT TCA AGC TCG CA	43,640–44,701	43,705–44,585	

**F-**ATC CTC CGG TGG TTG TTG AT	Chromosome	Chromosome	100%
**R-**AAC GTT TGA CAC CAC TGC TC	1,220,689–1,221,824	1,220,800–1,221,760	

**F-**CAG GTG GGA CGA ACT CTT CT	Chromosome	Chromosome	100%
**R-**CTC CTG TCC TGT TGC AAA CC	2,409,276–2,410,209	2,409,328–2,410,147	

**F-**CTT CGA ATC TGT TGC AGC GA	Chromosome	Chromosome	100%
**R-**GCG ACA CTG ATG CCT ACA AG	2,794,994–2,795,848	2,795,033–2,795,834	

**F-**TTT ACA ACC GCT GCT CCA AC	Chromosome	Chromosome	99%
**R-**GGA GGG GTA CGT GAT GGT AG	3,170,935–3,171,943	3,171,004–3,171,890	(WGS)

**F-**CGA GTG TCT GGT TTG CAA CA	Chromosome	Chromosome	100%
**R-**ATG GTA GGG GTT GTT GGA GG	3,671,665–3,672,574	3,671,778–3,672,460	

**F-**CGC CTC ACT TAA AGA ACC GG	Chromosome	Chromosome	100%
**R-**TTC CGT CGG TTA GTG GTT CA	4,702,142–4,703,114	4,702,215–4,703,038	

**F-**GAG TTA AGT GGA GGG GAG CA	Chromosome	NA	
**R-**TGT GAG CAT CGT CCT GTG TA	683,264–686,661		

**F-**TGG CAT CCA ACG ACA ATT GT	Chromosome	NA	
**R-**AGA GGC ATT CGA AGT TGA AGT	1,722,582–1,725,596		

**F-**ACC TGC GTG CTA AGT AAC CT	Chromosome	NA	
**R-**AGT GGA TGC TAC AAG GCT CA	4,001,142–4,004,064		

**F-**TAA GAT GAT GGG AGG AGC GG	Plasmid	Plasmid	100%
**R-**GAT TGC CAC TGT TTG CTT GC	675–1233	685–1,219	

**F-**CTG AAA ATG CAG CCG TTG AT	Plasmid	Plasmid	100%
**R-**TGC AAA TAG TAC CGT TGG CA	8,101–8,757	8,113–8,743	

**F-**GAC GAT GCC AGT GAA ACG AT	Plasmid	Plasmid	100%
**R-**ACG GTC ATT GCT CCT GAA GA	14,519–15,129	14,536–15,115	


**FIGURE 5:**
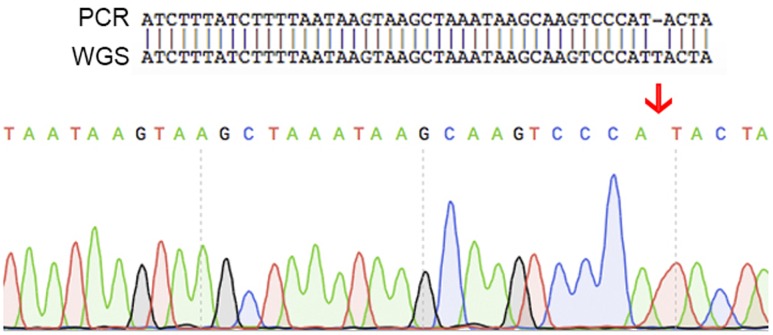
**A schematic showing the single nucleotide difference found between the sequence of PCR products and the whole genome sequencing data.** This difference was caused by unclear sequence signal of the PCR product. These results indicate that the whole genome sequence is of high quality.

An additional set of seven oligonucleotide primers were designed to target random positions across the circular chromosome together with a further three located on the plasmid (**Table 1**). All amplicons were successfully aligned to the genome with 100% identity. One of these contigs demonstrated a single nucleotide polymorphism (**Figure 5**). The raw sequencing data showed that this was most likely caused by an unclear sequencing signal from the amplicons produced. These validation checks supported the quality of the assembled genome for *C. estertheticum* DSM 8809.

During genome annotation, a total of 4,391 CDS were predicted along with 91 tRNAs, 47 rRNAs, 76 ncRNAs and a further 24 CDS were identified on the plasmid pDSM8809. No CRISPR sequences were identified in the *C. estertheticum* DSM 8809 genome, by Prokka. Nonetheless a 109-bp putative CRISPR was located using CRISPRfinder (Grissa et al., 2007) on the chromosome at position 803654–803763, wherein a hypothetical protein coding sequence was identified at this locus (**Table 2**).

**Table 2:** General comparative analysis of *C. estertheticum* DSM 8809; *Clostridium botulinum* A str. Hall and *Clostridium perfringenes* str. 13.

Features	*C. estertheticum* DSM 8809	*C. botulinum* A str. Hall	*C. perfringens* str. 13
Genome size (bp)	4,760,574	3,760,560	3,031,430
No. of plasmids	1	0	1
G+C content (%)	30.92	28.18	28.57
No. of predicted coding sequences (CDS)	4,391	3,397	2,670
No. of rRNA operons	47	24	30
No. of tRNA operons	91	81	98
No. of ncRNA operons	76	64	56
No. of phages (PHAST)	One intact	Three incomplete	One incomplete
	One incomplete		
	One questionable		
No. of CRISPR regions	0	4	0


To assess the metabolic potential of *C. estertheticum* DSM 8809, draft metabolic networks were generated using ORFs predicted by RAST annotation and KEGG in comparison with two other *Clostridium* species, namely *botulinum* str. Hall and *perfringens* str. 13 (**Table 2**). In *C. estertheticum* DSM 8809, a total of 2,003 of 4,504 ORFs were mapped to KEGG pathways. This number was higher than what those observed for *C. botulinum* str. Hall (1,608 mapped genes) or *C. perfringens* str. 13 (1,385 mapped genes) and which also mapped to KEGG. Among these, enzymes required to utilize various carbon-based sources including arabinose, cellobiose, galactose, inositol, maltose, mannose, melibiose, raffinose, rhamnose, ribose, salicin, starch, and sucrose were identified. This finding broadly supports the biochemical profiling of *C. estertheticum* DSM 8809, as reported earlier by Spring et al. (2003) and highlights the saccharoltyic capacity of this bacterium. Further, enzymes identified in *C. estertheticum* DSM 8809 and that were mapped to KEGG, and which are required for utilizing arabinose, cellobiose, maltose and rhamnose, were absent in either *C. botulinum* str. Hall or *C. perfringens* str. 13.

When considering the further metabolism of pyruvate, pathways required for its biochemical transformation resulting in the production of non-gaseous fermentation products including butyrate, acetate, lactate, and others were also identified.

### Antibiotic Genes Annotated in the Genome

No acquired antimicrobial resistance genes were identified by ResFinder 2.1 (Zankari et al., 2012). Prokka predicted a number of genes that encoded resistance to several antimicrobial compounds, including biocides and these are summarized in **Table 3**. Additional biocide and metal resistance genes were predicted using BacMet (Pal et al., 2014) and these results are also included in **Table 3**. Three putative prophage regions were predicted by PHAST (Zhou et al., 2011), among which a 31.5 kbp region (3,000,409–3,031,979) and a 19.1 kbp (3,034,721–3,053,909) region were located very close to each other. The third prophage region of 59.2 kbp in length (3,504,461–3,563,662) identified contained a predicted transposase gene from Tn*916*, together with a predicted *cusS* gene related to copper (Cu) and silver (Ag) resistance.

**Table 3:** Antibiotic/biocide/metal resistance genes identified following comparison of the genome sequence of *C. estertheticum* DSM 8809 with content located in selected databases.

Gene	Location	Resistant to
*alu*	Chromosome	Aluminum (Al)


*arsB*	Chromosome	Arsenic (As)


*arsD*	Chromosome	Arsenic (As)


*arsR*	Chromosome	Arsenic (As)


*bmr3*	Chromosome	Norfloxacin, puromycin, and tosufloxacin


*cadA*	Chromosome	Cadmium (Cd), zinc (Zn)


*copA*	Chromosome	Copper (Cu)
*corA*	Chromosome	Magnesium (Mg), cobalt (Co), nickel (Ni), manganese (Mn)
*corC*	Chromosome	Cobalt (Co), magnesium (Mg)
*crcB*	Chromosome	Camphor
*csoR*	Chromosome	Copper (Cu)
*cutC*	Chromosome	Copper (Cu)
*dnaK*	Chromosome	Copper (Cu)
*drrA*	Chromosome	Daunorubicin and doxorubicin
*fabK*	Chromosome	Triclosan
*fieF*	Chromosome	Iron (Fe), zinc (Zn), cobalt (Co), cadmium (Cd), nickel (Ni)
*marR*	Chromosome	Chloramphenicol and tetracycline
*merR1*	Chromosome	Mercury (Hg)
*mdtA*	Chromosome	Deoxycholate and novobiocin
*mdtB*	Chromosome	Deoxycholate and novobiocin
*mdtK*	Chromosome	Acriflavine, benzalkonium, chloramphenicol, deoxycholate, doxorubicin, ethidium, fluoroquinolones, fosfomycin tetraphenylphosphonium ion (TPP), and trimethoprim
*mntH*	Chromosome	Manganese (Mn), iron (Fe), cadmium (Cd), cobalt (Co), zinc (Zn)
*mntP*	Chromosome	Manganese (Mn), magnesium (Mg), methyl viologen, hydrogen
		Peroxide (H_2_O_2_)
*modB*	Chromosome	Tungsten (W), molybdenum (Mo)
*norM*	Chromosome	Acriflavine, ciprofloxacin, daunomycin, doxorubicin, ethidium bromide, kanamycin, norfloxacin, ofloxacin, and streptomycin
*ohrR*	Chromosome	Organic hydroperoxide
*pstB*	Chromosome	Arsenic (As)
*qacA*	Chromosome	Antiseptic and disinfectant compounds, e.g., intercalating dyes, diamidines, and quaternary ammonium salts
*ruvB*	Chromosome	Cetylpridininium chloride (CPC), chromium (Cr), dodine and 2-nitroimidazole
*stp*	Chromosome	Spectinomycin and tetracycline
*sugE*	Chromosome	Quaternary ammonium compound
*tet*(O)	Chromosome	Tetracycline
*vanW*	Chromosome	Vancomycin
*vmrA*	Chromosome	4,6-diamidino-2-phenylindole (DAPI), acriflavine, Ethidium
		Bromide, tetraphenylphosphonium (TPP), quaternary ammonium
		Compounds (QACs)
*ziaA*	Chromosome	Zinc (Zn)
*zupT*	Chromosome	Zinc (Zn), iron (Fe), cobalt (Co), nickel (Ni), copper (Cu), Cadmium (Cd)
*tehB*	Plasmid	Tellurite


Structural variations (SV) within the raw sequence reads across the genome were identified using Sniffles 0.1.0^1^. In summary 81 deletions, 4 duplications, 588 insertions, and 43 inversions were found in the chromosome, while 1 deletion, 1 duplication, 13 insertions, and no inversion were found in the plasmid.

The 23,034-bp plasmid pDSM8809 was mainly composed of genes of unknown function. Those that could be identified included a predicted transposon Tn*3*-like resolvase-encoding *tnpR*. Interestingly, one of the deletions identified by SV analysis included the *tnpR* gene (see **Figure 2**). The plasmid was also predicted to carry a *tehB* gene that is known to contribute to tellurite resistance.

### Virulence Genes Annotated in the Genome Sequence

*Clostridium estertheticum* is not known to be pathogenic to human health. To determine whether or not any virulence factors may be present, the genome was searched using BLASTP and the virulence factor database (VFDB) (Chen et al., 2011).

**FIGURE 6:**
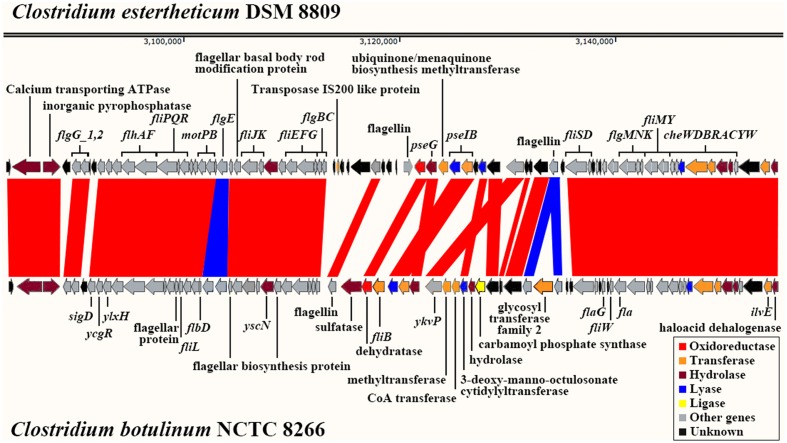
**A representation showing the alignment of flagella-related gene clusters comparing those identified in *C. estertheticum* DSM 8809 with similar loci in *Clostridium botulinum* NCTC 8266.** Each arrow shown represents for a single CDS.

Based on this output 54 potential virulence factors were identified in the genome. For example, several flagella-related genes were identified and located on the *C. estertheticum* DSM 8809 genome. A similar cluster was also identified in *C. botulinum* NCTC 8266 (Accession number: NZ_CP010520) with a number of notable differences and illustrated in **Figure 6**. In particular, it was noted that there was an area where the genes have been reordered and which was flanked by two conserved loci. Most genes in this particular location are coded on the negative strand. The less conserved region consisted of genes coding for flagellin, a component of bacterial flagella, and included the *pseGIB* genes whose function is to modify flagellin. *C. estertheticum* DSM 8809 also possessed an insertion sequence denoted as IS*200* and which was absent from the genome of *C. botulinum* NCTC 8266. In contrast, the *fliB* gene identified in the latter bacterium was not found in *C. estertheticum* DSM 8809. Genes upstream and downstream of this region were found to be more conserved between these organisms. As an example of this, a group of flagellar related genes including *flgBC. fliEFGJKLPQR. flbD. motPB. flhAF* were located upstream whilst *fliSDMY. flaG. cheWDBRACYW* located downstream.

PathogenFinder 1.1 (Cosentino et al., 2013), a program that runs comparisons between pathogenic and non-pathogenic bacteria using whole genome sequence data, showed that, when *C. estertheticum* DSM 8809 was used as the query sequence, results demonstrated that it was non-pathogenic with an accuracy of 88.6%.

## Discussion

The complete genome sequence of *C. estertheticum* DSM 8809 was determined. Following SMRT^®^ sequencing, the genome was assembled, into a single contig of 4,760,574 bp representing the bacterial chromosome along with a smaller contig of 25,039 bp representing a plasmid pDSM8809. Compared with other members of this bacterial genus such as *C. botulinum* and *C. perfringens. C. estertheticum* harbors a larger genome with a marginally higher GC content.

Enzymes that contributed to the metabolism of carbon-sources supporting the saccharolytic phenotype of this bacterium were identified. Furthermore, pathways contributing to end-products of anaerobic metabolism, including butyrate, acetate, and lactate among others were noted. These observations support earlier work describing the biochemical profile of *C. estertheticum* DSM 8809.

The genome was queried in an effort to identify genes potentially important to public health, namely antibiotic/biocide/metal resistance-encoding genes and virulence factors based on sequence similarity. Our analysis identified a flagellar-related gene cluster, multiple genes potentially related to antibiotic, biocide and metal resistance, along with several predicted virulence factor genes, and a transposase-encoding gene on the plasmid. Besides the findings above, the high quality complete genome sequence generated in this study also forms an important basis for further studies that will lead to a better understanding and control of BPS. Use of the genes annotated in this genome may prove useful for the selection of candidate diagnostic markers to be assessed for inclusion in molecular-based assays designed to facilitate the rapid discovery of the bacterium on vacuum-packed meat surfaces, thereby improving food quality and reducing economic loss.

## Author Contributions

ZY and SF designed the study. ZY, LG, EB, and RR participated in the lab work. ZY conducted the bioinformatics analysis with the help and supervising from PG and DH. PW, DB, and SF supervised the whole project.

## Conflict of Interest Statement

The authors declare that the research was conducted in the absence of any commercial or financial relationships that could be construed as a potential conflict of interest.

## Footnotes

1 https://github.com/fritzsedlazeck/Sniffles
